# Size Reduction in Early European Domestic Cattle Relates to Intensification of Neolithic Herding Strategies

**DOI:** 10.1371/journal.pone.0141873

**Published:** 2015-12-02

**Authors:** Katie Manning, Adrian Timpson, Stephen Shennan, Enrico Crema

**Affiliations:** 1 Institute of Archaeology, University College London, London, United Kingdom; 2 Research Department of Genetics, Evolution and Environment, University College London, London, United Kingdom; 3 CaSEs—Complexity and Socio-Ecological Dynamics Research Group, Universitat Pompeu Fabra, Department of Humanities, Barcelona, Spain; University College Dublin, IRELAND

## Abstract

Our analysis of over 28,000 osteometric measurements from fossil remains dating between c. 5600 and 1500 BCE reveals a substantial reduction in body mass of 33% in Neolithic central European domestic cattle. We investigate various plausible explanations for this phenotypic adaptation, dismissing climatic change as a causal factor, and further rejecting the hypothesis that it was caused by an increase in the proportion of smaller adult females in the population. Instead we find some support for the hypothesis that the size decrease was driven by a demographic shift towards smaller newborns from sub-adult breeding as a result of intensifying meat production strategies during the Neolithic.

## Introduction

Changes in animal body size have been shown to correlate with various ecological factors such as reproductive behaviour and environmental modifications including predator dynamics and rising temperatures [1–5]. Yet, whilst selection can be intense over short time scales i.e. a few generations, its direction may vary through time, cancelling out long-term evolutionary trends [6–9]. Accessing the sort of long-term datasets required to identify such diachronic trends, however, can be problematic due to taphonomic bias, gaps in the fossil record, etc. [10]

Archaeozoological assemblages meanwhile offer an intermediate time scale, providing potential insight into inter-generational phenotypic change and underlying evolutionary trends. Size reduction, for example, has long been recognised as a consequence of the domestication process [11–14] and several hypotheses have been proposed to explain the phenomena, namely deterioration in pasture conditions and early weaning [14], protection from predation and reduction in mobility [15].

It has been suggested that European cattle continued to reduce in size over the course of the Neolithic, Bronze Age, and pre-Roman Iron Age [11, 16], and this is well documented in several regional case studies [17–23]. Using archaeological data and more than 28,000 osteometric measurements, our results confirm a substantial and consistent reduction in domestic cattle size throughout the Neolithic at the sub-continental scale. We estimate the evolutionary rate of body size change as a function of time, demonstrating the long-term evolutionary development of early domestic cattle. For clarity, we use the term ‘evolution’ to include selective breeding, by considering humans as merely one of many species, thus removing the somewhat philosophically flawed distinction between ‘artificial’ and ‘natural’ selection.

We consider a number of hypotheses, which have previously been proposed to explain the observed trend, and we specifically test two of them:

1. The reduction in adult size merely reflects an increase over time in the ratio of the smaller female adults, as a consequence of changing herding strategies, such as an intensification of dairying practices.
2. The reduction in adult size reflects a shift in the age distribution of the cattle population towards a younger sub-adult reproduction age, causing the offspring to achieve smaller adult size due to the physiological and morphological constraints of the mother giving birth before having reached adult body size. This phenomenon has been well documented in the sheep of St Kilda [2–3].

## Materials and Methods

### Data

This study adopts an inclusive approach to the data in order to formally test patterns in the published literature. As such we have not made judgements about the accuracy of species identification or measuring procedure [24–25], and have only excluded samples that researchers have identified as erroneous. In order to guarantee a minimum standardization in the measuring procedure we have applied the criterion of only using osteometric measurements from fully fused adult remains measured according to the von den Driesch [26] standard method. Despite potential errors in the original recording of these data, there is no reason to believe these errors would introduce a systematic bias, therefore this inclusive approach is inherently conservative since random errors in the data would only serve to add additional background noise to underlying trends.

**Fig 1:**
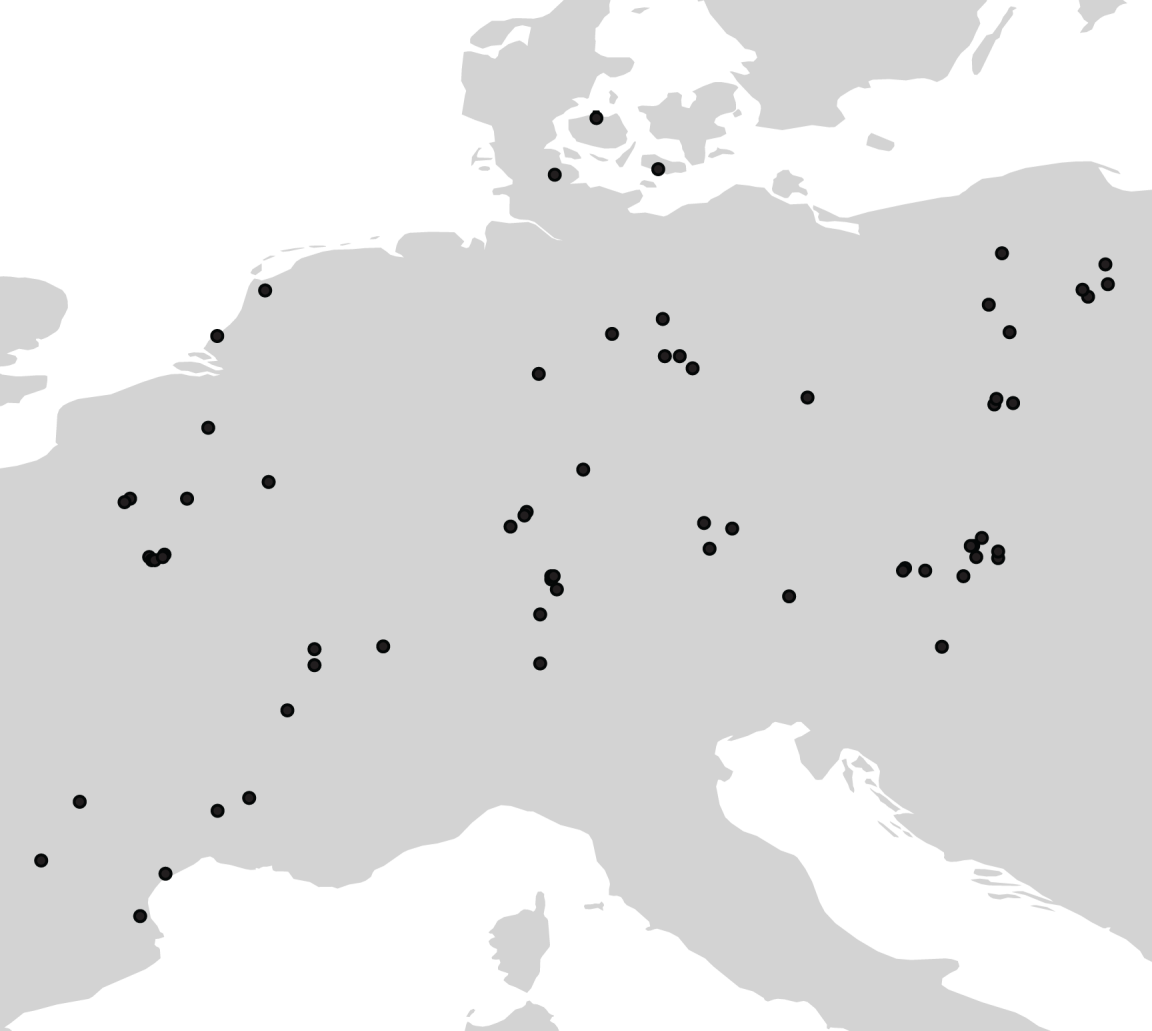
Map showing location of all 81 site phases used in the osteometric analysis.

Data from the British Isles were excluded, to avoid the potential bias of a selective pressure favouring smaller individuals for ease of sea transport. Finally, osteometrics with less than 10 measurements, and site phases with less than 10 osteometrics were excluded to reduce sampling noise, whilst still ensuring good skeletal, geographic and temporal representation, producing a total sample of 28,266 measurements from 152 postcranial and dental elements for *Bos taurus* (n = 16,568), *Bos primigenius* (n = 1119), *Sus s*. *domesticus* (n = 5021), *Ovis aries* (n = 3394), *Capra hircus* (n = 714), and *Canis familiaris* (n = 1450). These were obtained from 81 phases identified in 70 archaeological sites in central Europe (Fig 1), dating from the Early Neolithic to the Early Bronze Age (c. 5600–1500 BCE). Where available, bones have also been allocated a sex classification (male, female, and castrate) according to the original analyst’s determination in order to examine the size trend for males and females independently. We also utilise additional published data sex trend data and age profile data for *Bos taurus*. The sex trend data comprises 1340 counts of positively identified male, female and castrate bones, based on morphological criteria, from 38 site phases. The age profile data comprises relative proportions of different age groups from 116 site phases. All osteometric, chronological and age profile data were derived from the EUROEVOL database (for details on the project see http://www.ucl.ac.uk/euroevol/), which is publicly accessible at http://discovery.ucl.ac.uk/1469811/, whilst the sex trend data are provided as an independent csv. file in the SI (S1 Table).

### Transformation methods to combine metrics

Archaeological assemblages are typically characterised by only a few measurable bones, and often focus on a limited number of osteometrics, considerably restricting the sample size with which to statistically test hypothesised changes in animal body size. In order to overcome this problem, a number of different scaling methods have been proposed to combine different osteometrics (see Meadow 1999 [27] for a review). Although these techniques hinder the study of shape and proportion, which can be investigated through relative differences in osteometrics [28–29], they have the major benefit of generating large sample sizes that provide greater sensitivity in detecting and quantifying the size decrease as well as testing if the decrease is significant. Therefore, we employ a Log Size Index (LSI [30, 27]), which is calculated for each osteometric by dividing the measurements by their mean, then taking the logarithm. LSI takes into account differences in scale, enabling statistical comparison between different groups, and the aggregation of different osteometrics. The mean LSI per site phase for each species is reported in S2 Table.

### Chronological sequencing

We employ two dating methods for different aspects of our analyses. Firstly, in order to identify broad temporal trends we use a coarse-grained resolution, with all site phases being assigned to an archaeological ‘period’ i.e. Early Neolithic (c. 5600–4800 BCE), Middle Neolithic (c. 4800–3500 BCE), Late Neolithic (c. 3500–2500 BCE) or Early Bronze Age (c. 2500–1500 BCE). By using these chronological periods we assess the directional size change in cattle body size, the proportion of adult females in the population, and the proportion of different age groups.

Whilst categorising data into broad archaeological periods is a useful way of identifying an underlying trend, we also wanted to calculate the evolutionary rate of phenotypic change for comparison with different species, which required greater temporal resolution. We therefore developed a method that hierarchically selects from different sources of chronological evidence. At the highest level, we use the midpoint of the chronological range published in the site report, which often integrates a variety of evidence filtered through the expertise of the author, for example incorporating Bayesian analysis of both radiocarbon and stratigraphic evidence. If this was not available we generated a summed probability distribution from all radiocarbon dates for each site phase with more than five radiocarbon samples (available in the EUROEVOL database), then used the midpoint of the 95% (2-tails) confidence interval. The third level used the mean of the Gaussian date estimate for the archaeological culture associated with that phase [31]. Finally, if none of the above were available, we resorted to using the midpoint of the standard date range for that culture published in the literature (see Manning et al. 2014 [31] for a list of the standard date ranges used).

### Characterising size change

**Fig 2:**
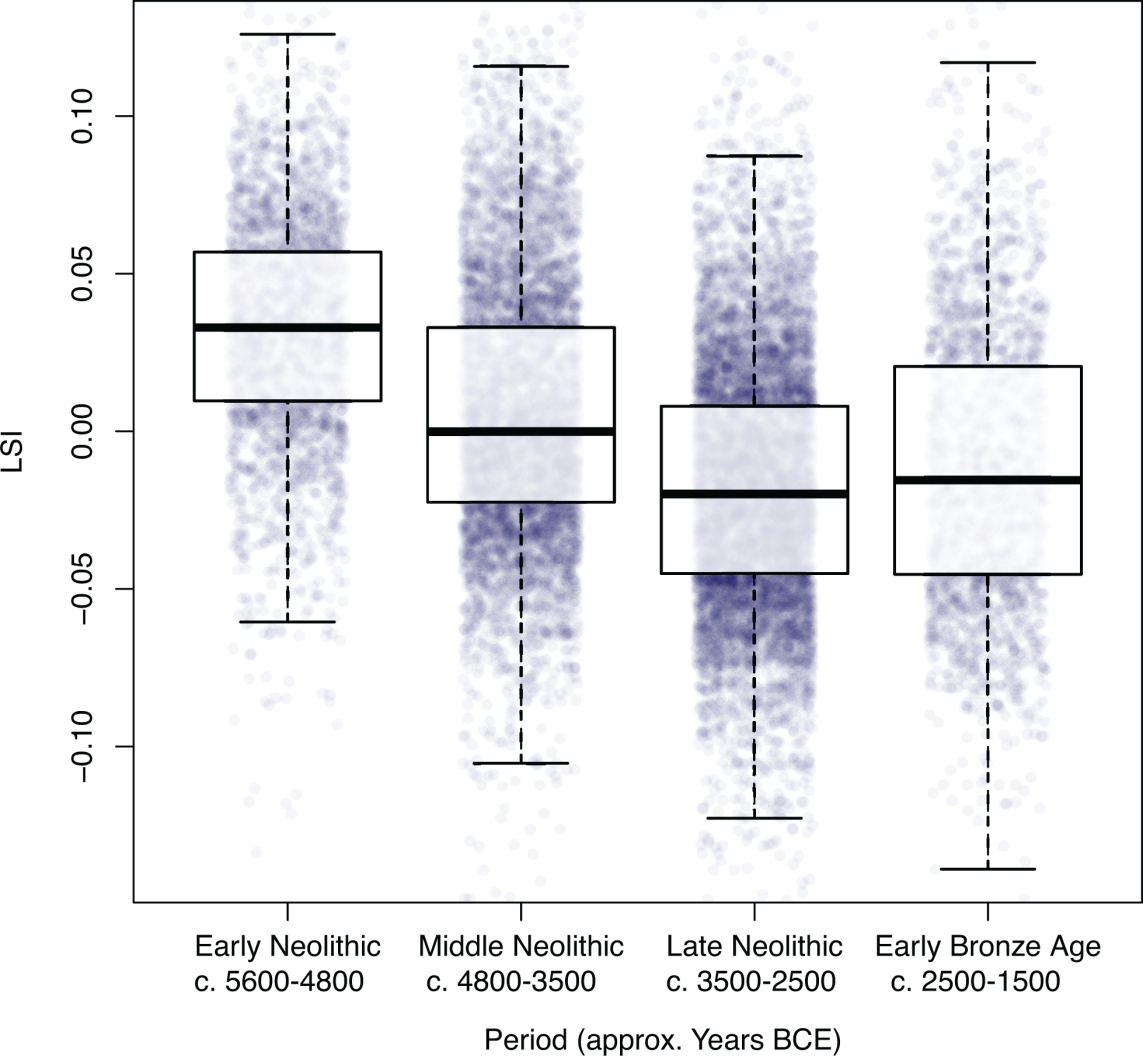
Full distribution and box-plot overlay of all LSI transformed measurements for *Bos taurus* categorized by broad chronological period. Each measurement is jittered to reveal the distribution of the data.

**Fig 3:**
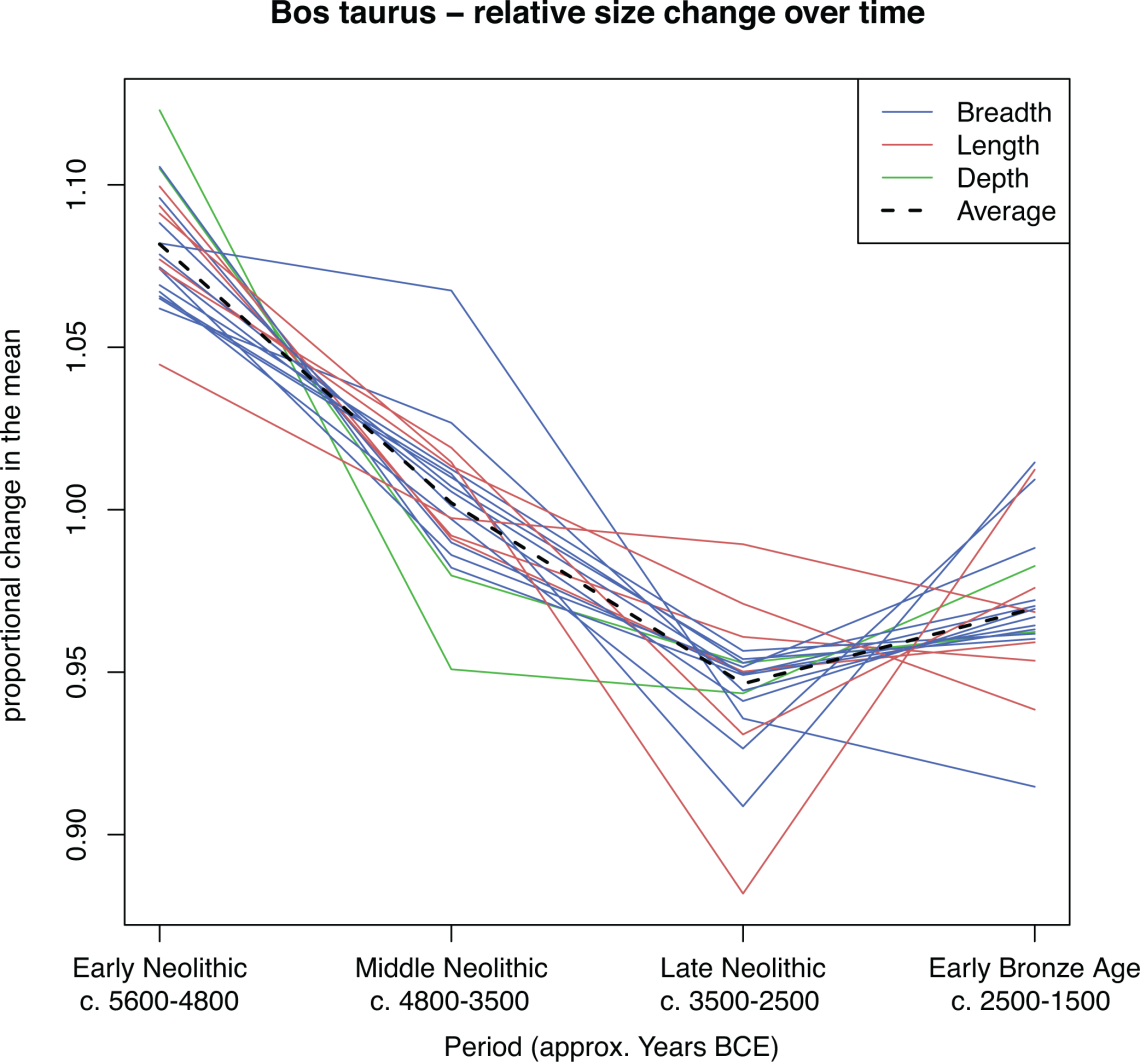
*Bos taurus* proportional change in the mean of the 20 best represented osteometrics coded according to axis. Blue lines represent breadth, red lines represent length and green lines represent depth. The dashed line is the mean value for all measurements. The raw osteometrics and their proportional change are listed in Table 2.

Using an ANOVA and a Tukey’s *post-hoc* multiple comparison test we examine the difference in the full distribution of LSI values between each of the four periods for *Bos taurus* (Table 1). We plot the full distribution of all LSI values for *Bos taurus*, across the four periods (Fig 2). We then quantify the overall change in cattle size through time after applying an inclusion criterion of >25 measurements per osteometric per site phase by using the mean raw measurement per osteometric for each period, which is then divided by the mean for all periods (Table 2). We refer to this as the proportional change in the mean. Whilst mean values offer little in the way of demonstrating within period variability, they provide a useful tool for illustrating the degree of variation between periods. Each osteometric was then colour coded according to the three bone axes (length, breadth and depth), in order to evaluate potential allometric variation (Fig 3).

**Table 1:** Tukey HSD results showing difference in mean levels of Period for Bos taurus.

		95% quantile		
Period	Difference	Lower	Upper	p-value
EN-MN	-0.0266	-0.0295	-0.0237	<0.00001
MN-SLN	-0.0231	-0.0295	-0.0237	<0.00001
LN-EBA	-0.0069	-0.0099	-0.0039	<0.00001

**Table 2:** Mean measurement (in mm) per osteometric for each period, n = number of measurements per osteometric, the proportional change in the mean between periods is shown in parentheses. This is also averaged for breadth, length and depth measurements, and reported for each period (note, Scapula GLP and Scapula LG have been grouped with the length osteometrics according to von den Driesch’s original anatomical justification, although they could arguably be considered with the breadth osteometrics).

Osteometric	n	Early Neolithic	Middle Neolithic	Late Neolithic	Early Bronze Age
Astragalus Bd	561	46.58 (1.10)	42.07 (0.99)	40.36 (0.95)	40.98 (0.96)
Humerus Bd	266	87.18 (1.06)	84.30 (1.03)	77.53 (0.94)	79.39 (0.97)
Humerus BT	248	80.86 (1.07)	75.67 (1.01)	71.44 (0.95)	73.02 (0.97)
Metacarpal Bd	333	64.22 (1.07)	60.01 (1.00)	55.76 (0.93)	60.74 (1.01)
Metacarpal Bp	310	63.14 (1.07)	59.88 (1.01)	56.49 (0.95)	57.64 (0.97)
Metatarsal Bd	388	61.06 (1.11)	54.24 (0.98)	52.42 (0.95)	53.20 (0.96)
Metatarsal Bp	327	51.43 (1.07)	47.21 (0.99)	45.56 (0.95)	47.31 (0.99)
PH1 Bp	355	32.71 (1.08)	32.26 (1.07)	28.28 (0.94)	27.65 (0.91)
PH2 posterior Bp	296	31.16 (1.07)	29.56 (1.01)	26.57 (0.91)	29.67 (1.01)
PH3 MBS	211	26.07 (1.07)	24.69 (1.01)	23.33 (0.96)	23.45 (0.96)
Radius Bp	262	88.45 (1.08)	82.60 (1.00)	78.24 (0.95)	78.75 (0.96)
Tibia Bd	387	67.96 (1.09)	62.52 (1.00)	58.77 (0.94)	60.54 (0.97)
Average Breadth prop.	5262	1.08	1.01	0.94	0.97
Astragalus GLl	586	73.68 (1.09)	66.84 (0.99)	64.74 (0.96)	64.25 (0.95)
Astragalus GLm	426	67.27 (1.10)	60.64 (0.99)	58.13 (0.95)	58.68 (0.96)
PH1 GLpe	328	66.20 (1.08)	62.29 (1.01)	59.69 (0.97)	57.69 (0.94)
PH3 Ld	173	58.99 (1.04)	56.32 (1.00)	55.87 (0.99)	54.69 (0.97)
Scapula GLP	233	71.09 (1.09)	66.10 (1.01)	57.45 (0.88)	65.95 (1.01)
Scapula LG	216	60.30 (1.07)	57.21 (1.02)	52.26 (0.93)	54.79 (0.98)
Average Length	2294	1.08	1.00	0.95	0.97
Astragalus Dl	359	41.26 (1.11)	36.59 (0.98)	35.58 (0.95)	35.94 (0.96)
Astragalus Dm	283	41.35 (1.12)	35.01 (0.95)	34.74 (0.94)	36.18 (0.98)
Average Depth	642	1.11	0.97	0.95	0.97

### Calculating the rate of phenotypic change

**Fig 4:**
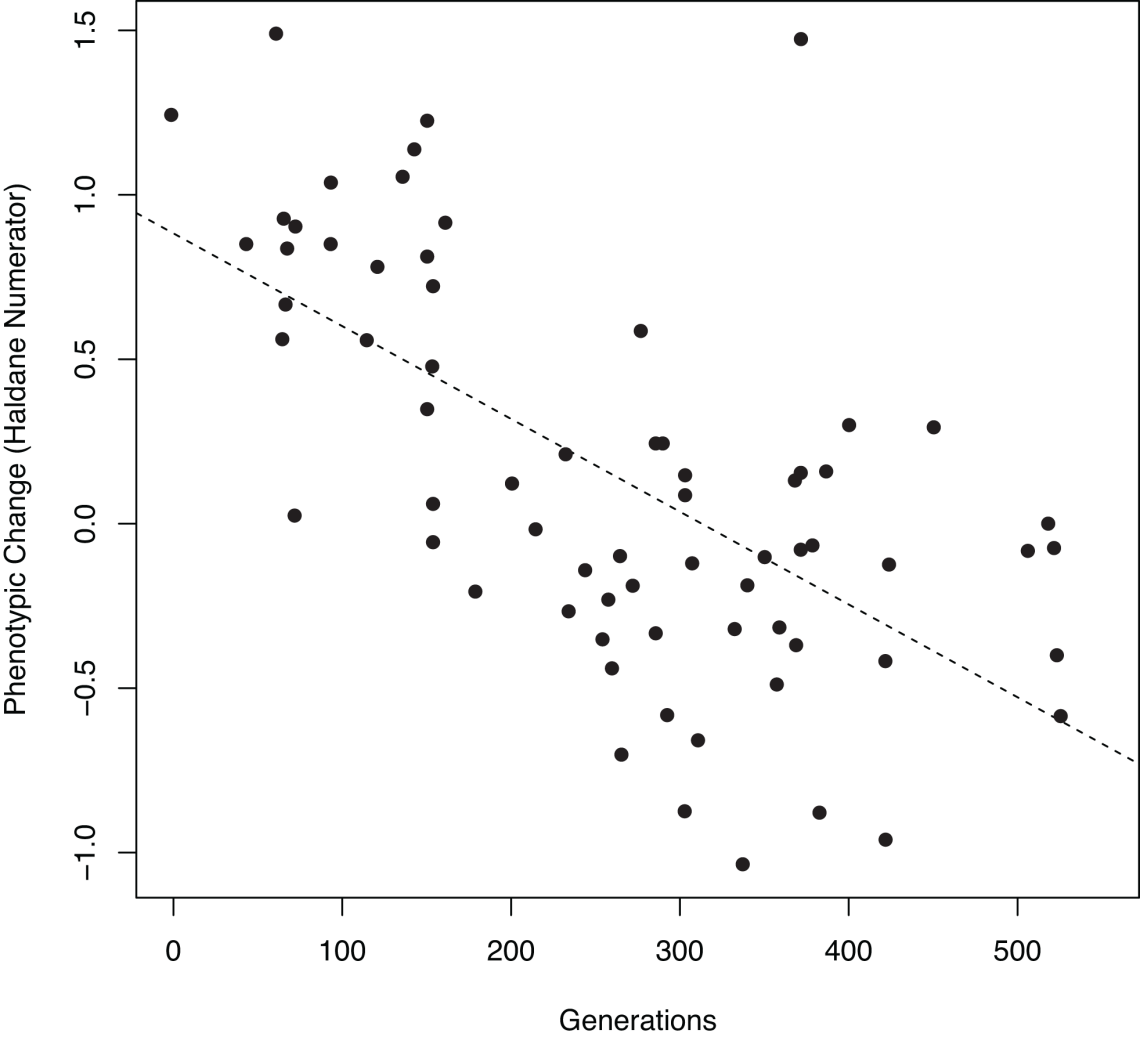
Evolutionary rates for Neolithic cattle, showing the Haldane numerator against time expressed in number of generations.

Using the fine-scale chronological data, we calculated evolutionary rates for all domestic species and *Bos primigenius*, in haldanes (h), across the period Early Neolithic to Late Neolithic. This was achieved by fitting a least-squares linear model of the ratio between the mean LSI and the pooled standard deviation (known as the Haldane numerator) against time expressed in number of generations [32]. In order to calculate the evolutionary rate of change for each species we used the following generation times: *Bos taurus*– 7 years [33]; *Bos primigenius*– 7 years [34]; *Ovis aries*– 2 years [35]; *Capra hircus*– 2.5 years [36]; *Sus s*. *domesticus*– 5 years [37; *Canis familiaris*– 4 years [38]. The results (Fig 4 and Table 3) provide a measure of absolute change expressed in standard deviations per generation, and are comparable across different species [39–40].

**Table 3:** Haldane rates (x 10^3^) for Neolithic domestic animals and wild cattle (n = number of phases, g = generation time). See S2 Table for raw data.

Species	haldane	StdE	R^2^	P value	n	Start (BP)	End (BP)	g
*Bos taurus*	-2.8	0.4	0.40	<0.0001	69	5560	1875	7
*Bos primigenius*	-0.9	0.8	0.09	0.266	15	5100	2971	7
*Ovis aries*	0.3	0.2	0.09	0.175	23	5675	1875	2
*Capra hircus*	-0.4	0.4	0.10	0.348	11	5675	1890	2.5
*Sus s*. *domesticus*	0	0.6	<0.01	0.993	34	5250	1890	5
*Canis familiaris*	1.4	1.0	0.17	0.164	14	4480	2010	4

#### Estimating changes in the demographic structure of cattle herds`

**Fig 5:**
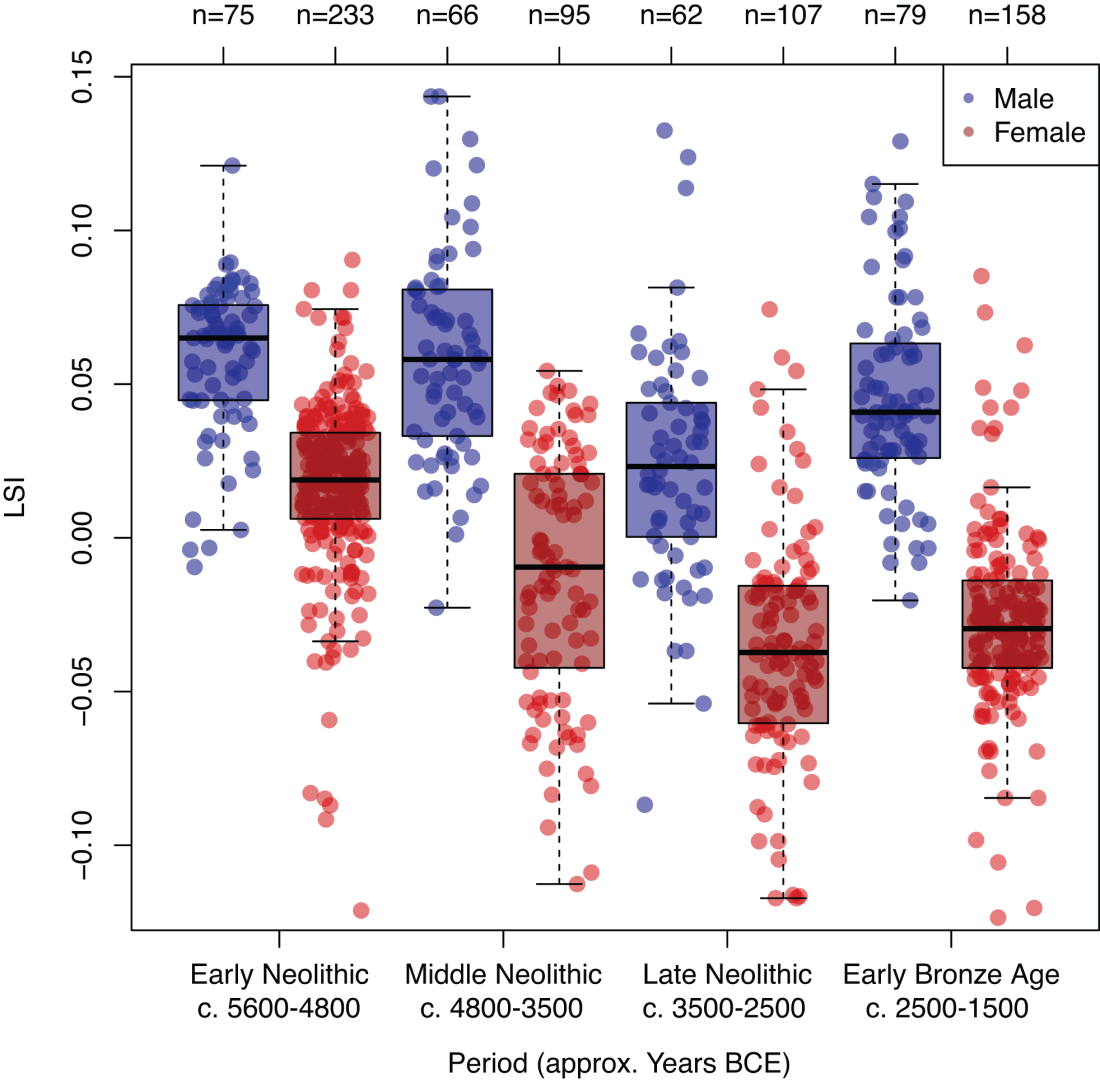
Full distribution and box-plot overlay of all LSI transformed measurements for *Bos taurus* male (n = 282) and female (n = 593) categorized by broad chronological period, revealing a synchronous trend (n = number of osteometric measurements per sex per period).

Our sex trend data, comprising 1340 counts of positively identified male, castrate, or female *Bos taurus* remains, were based on the original analysts’ morphological assessment of long bones and horncores, from 38 site phases (S1 Table). Castrate counts were ignored due to poor representation since they were only present in 7 phases (18%), which may be a result of differential recording practices rather than any genuine presence or absence. We then estimated the proportion of adult females during each period using a beta distribution with a uniform prior, with the shape parameters α = count of adult females + 1, and β = count of adult males +1, to take into account uncertainty in the true proportion when sample sizes are small (Fig 5).

**Fig 6:**
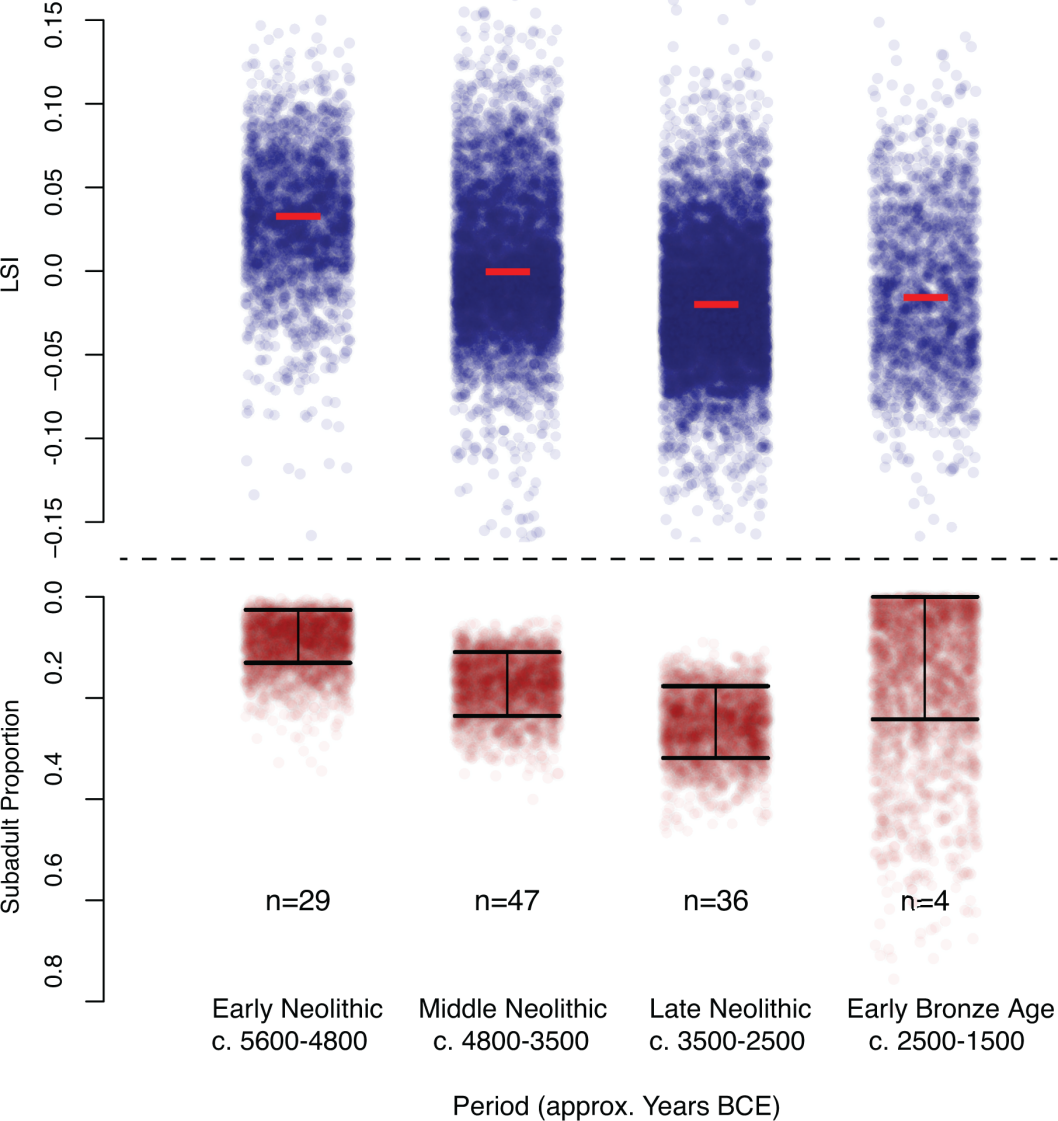
Top: LSI for all LSI transformed measurements (each blue dot) for adult *Bos taurus*; horizontal jittering is merely to aid viewing. Red bar indicates the median. Bottom: relative proportion of site phases with predominantly sub-adult remains over time. Sample sizes shown are the total number of site phases in each period. Red dots are random samples from each beta distribution, and are merely to aid viewing the uncertainty in the proportion estimates. Black bars indicate the 75% highest posterior density.

There are a variety of techniques for determining the age-at-death of animals derived from archaeological contexts, including epiphyseal fusion, tooth eruption and wear sequences, cranial sutures and antler or horn development. Due to the varying quantification methods of these different techniques and the diversity of age groups used by different researchers, we have categorised each of the 116 site phases into either ‘predominantly sub-adults (1–3 years)’ or not (all other age groups, including no age trend, neonates, juveniles (1–12 months) and adults (>3 years). The key distinction is between juveniles who are too young to reproduce, sub-adults who are morphologically still immature but are able to reproduce, and adults who have reached maturity, have fully fused bones, and contribute to the osteometric data. These categories were assigned based either on the general trend observed by the original analyst or by binning the raw count data provided in original reports into the respective age brackets of each category. The data were then used in two distinct analyses in order to investigate our second hypothesis. Firstly, we counted the number of site phases in each broad temporal period that comprised predominantly ‘sub-adult’ and ‘other’. The change over time was tested for significance using a Chi-squared test. Furthermore, we used these counts as the shape parameters in a beta distribution in order to generate estimates of the proportion of sub-adults in the population, allowing for the uncertainty of small sample sizes (Fig 6).

## Results

### Directional size change in cattle body mass

Fig 2 and Fig 3 illustrate a clear directionality in size decrease for Neolithic cattle, with an apparent uptick towards the beginning of the Early Bronze Age. Results from the Tukey test demonstrate that *Bos taurus* LSI are significantly different between all periods (p<0.00001), with a total difference in the mean LSI between the Early and Late Neolithic of -0.0497 (Table 1).

Fig 3 and Table 2 show all 20 *Bos taurus* osteometrics with more than 25 measurements per period, revealing a synchronous decrease between the Early and Late Neolithic of 12.6% (mean) varying between 5.3% and 19.2% size decrease. This trend appears to slightly reverse towards the Early Bronze Age as some osteometrics show an increase (max = 14.8%), whilst others continue to decrease (max = 3.4%). On average this gives an overall increase of 2.8%, although without data from subsequent Bronze Age periods it is unclear whether this is the start of a directional upward trend.

Each osteometric is one-dimensional, however both mass and size (volume) are proportional to the cube of these metrics, since they are 3-dimensional. Therefore an average linear reduction of 12.6% equates to 1 - (1–0.126)^3^, giving a reduction in size and mass of 33.2%. This assumes the shape of cattle remained approximately similar, which is supported by separate calculations for mean breadth reduction (32.9%) and mean length reduction (32.4%). However we were also able to estimate absolute limits in the size decrease given the total volume must be a function of the combination of linear osteometrics. It is not required for this function to be known since the total decrease must be greater than the smallest linear decrease cubed, and also smaller than the greatest linear decrease cubed. This provides absolute limits of 15.1% to 47.2%.

Although analysis of the allometric changes characterising Neolithic cattle populations is beyond the scope of this paper, the separation of breadth, length and depth measurements provide some indication of changes in body proportions over time and would clearly benefit from a more detailed study that takes into account the effects of sexual dimorphism, as well as regional complexities in bone allometry.

### Rates of Phenotypic change

Since the trend of size reduction during the Neolithic appears to slightly reverse at the Early Bronze Age, the Haldane evolutionary rate was only calculated across the Neolithic using the data at the scale of individual phases (Table 3; Fig 4). Only *Bos taurus*, and no other domestic taxa, showed a significant change through time (-2.8±0.4 haldanes x 10^3^, p<0.00001, number of phases = 70). Therefore, whilst other species may have undergone more regionally and temporally sensitive changes in size, they do not demonstrate the same scale of directional size change observed in cattle.

### Demographic changes to cattle herds

Using the independent sex data as shape parameters in a beta distribution, we estimated the most likely proportion of adult females in each period, and the 95% confidence intervals to reflect the uncertainty from small samples sizes. These results show a slight decrease in the proportion of adult females from 0.70 (95% HPD = 0.65–0.75) during the Early Neolithic to 0.62 (95% HPD = 0.58–0.65) by the Late Neolithic (Table 4). The lack of overlap between the 95% HPD indicates a statistically significant decrease in the proportion of adult females, corroborated by a Chi-squared test (p = 0.0075).

**Table 4:** Raw counts of adult male and adult female bones identified according to morphological criteria (n = number of phases). The proportion values and 95% CI are calculated from the beta distribution.

Period	n	Males	Females	Proportion	95% HPD
Early Neolithic	7	99	244	0.70	0.65–0.75
Middle Neolithic	7	71	191	0.72	0.66–0.77
Late Neolithic	20	228	379	0.62	0.58–0.65
Early Bronze Age	4	25	45	0.63	0.52–0.73

Furthermore an assessment of LSI measurements, which had been positively identified as male (n = 282) or female (n = 593) also shows that both sexes undergo synchronous size change. T-tests show a decrease of 0.035 in the mean LSI of males between the Early and Late Neolithic (p < 0.00001), and in females an even greater decrease of 0.053 (p < 0.00001) across the same period (Fig 5). The results of our analysis therefore contradict the hypothesis that the reduction in cattle size was attributable to an increase in the proportion of adult females in the overall population.

Our analysis of the age profile data shows an increase in the proportion of sub-adults, with the proportion of site phases with predominantly sub-adult remains rising from6% during the Early Neolithic to 15% in the Middle Neolithic and 31% in the Late Neolithic, which is synchronous with the decline in average adult cattle size. The proportion of site phases with predominantly sub-adult remains then decreases during the Early Bronze Age, synchronous with the uptick in cattle size (Fig 6). Chi-squared test shows a statistically significant difference in the proportion of site phases with predominantly sub-adult remains, between the Early and Late Neolithic (p = 0.0132).

## Discussion

### Summary of results

It has long been recognised that European domestic cattle reduced in size over the course of the Neolithic, and previous studies have demonstrated this trend at different regional and chronological scales e.g. north/central Europe [11, 16], the Paris Basin [21], Poland [22] and Switzerland [23]. Our analysis evaluates size change at a much broader temporal and spatial scale, and our results strongly support this trend, demonstrating a substantial reduction in domestic cattle size but not any other domestic species during the European Neolithic. This may appear to contradict recent studies, which have demonstrated a size reduction in other taxa, for example pig [41], although this apparent discrepancy is likely due to the spatial and temporal scale of the different analyses. Whilst other domestic taxa may have undergone regional or discontinuous variation in body size, they do not exhibit the same sort of long-term and geographically widespread trend observed in *Bos taurus*. This suggests that only cattle were subject to the sort of consistent evolutionary pressure that resulted in such a directional phenotypic change.

Our results suggest a substantial reduction in body mass of c. 33% in only 3100 years. This is supported by the high evolutionary rate estimated from our data (-2.8±0.4 haldanes x 10^3^), which can be fairly compared with Purugganan and Fuller’s [32] haldane rate of change for plant domestication traits. They report a rate of 1.3±0.2 haldanes x 10^3^ for barley (*Hordeum vulgare*) and 0.9±0.2 for einkorn wheat (*Triticum monococcum*), which is of the same order of magnitude as our results and suggests a strong selective pressure acting on Neolithic cattle.

A number of different hypotheses have been proposed to explain a reduction in livestock body size, which include: a reduction in mobility, reduced nutritional levels [16], and a reduction in sexual dimorphism, which is a well-studied consequence of the domestication process [42–43]. In the following section we rule out certain proposed causal factors and test two specific hypotheses, namely an increase in the proportion of the smaller females, and a decrease in the reproduction age.

### Ruling out possible causes: Domestication, introgression and climate change

Size reduction and the development of other pedomorphic or neotenic features have long been recognised as consequences of the domestication process [44–46, 42]. However, the core package of domestic animals (cattle, sheep, goat, and pigs) were domesticated in the northern Levant during the 10^th^-9^th^ millennia BCE [43, 46] and subsequently exported to Europe [47]. Hence the size reduction reported here post-dates the domestication process by more than 3000 years, suggesting a phenotypic adaptation distinct from those associated with domestication. An important cause of initial size reduction during domestication, for example, is a decrease in sexual dimorphism amongst early domesticates. Whilst this has been clearly demonstrated in Early Neolithic contexts in the Middle Euphrates [43], it is still possible that the process of decreasing sexual dimorphism continued as domestic cattle were more intensively exploited over the course of the Neolithic. However, this does not appear to have driven the size reduction observed in early European cattle, as our results show a significant parallel size change in both male and female domestic cattle. A decrease in sexual dimorphism over the course of the Neolithic would result in the distribution of the two sexes becoming more similar by the Late Neolithic, but our results show that this sexual dimorphism was maintained. Furthermore, the size reduction associated with proto-domestication is also observed in Near Eastern sheep [13] goat [48] and pigs [12, 49–50], and yet in Europe these species do not undergo a directional size decrease over the course of the Neolithic. Therefore our results suggest that the observed size decrease in Neolithic cattle was distinct from the initial process of domestication.

In some circumstances introgression with wild cattle may offer some explanation for a size change. Male aurochs were much larger than domestic bulls, and therefore introgression provides a potential explanation for the opposite trend of a size increase. Even if introgression was initially prevalent and became less common over time, we would expect to see the rate of size increase gradually retard, until the size remained approximately constant through time (subject to random drift), but certainly not a size decrease. Furthermore, recent aDNA work corroborates the importation of Near Eastern cattle stock [51], and provides little evidence for a genetic contribution of native aurochs to the domestic gene pool [52–53].

Another possible explanation is that an exogenous force, such as climate, was the underlying cause of the observed size reduction. Bergmann’s rule [54] for example, predicts that populations living in warmer environments will comprise smaller individuals than those from a colder environment. However, we would expect an exogenous force, such as climate to have a similar effect on all species, both domestic and wild. Our analysis shows the directional size reduction only affected cattle, and therefore we reject climate as a causal factor.

### So, why did cattle undergo such a substantial size reduction?

Given the expectation that farmers might improve meat yields by increasing the body mass of their livestock, or increase the number of larger males for traction, it is surprising to observe such a dramatic reduction in average body mass. Indeed, pre-industrial European cattle underwent at least one well-documented size increase during the initial period of the Roman conquest [55–59]. This is generally interpreted as a reflection of cattle improvement, linked either to an intensification of arable agriculture, in which more draft oxen were required [57], or the need for provisioning an increasingly consumer based economy [60]. Why Neolithic farmers apparently bred smaller cattle, but not their other domestic animals, is therefore an intriguing puzzle.

#### Hypothesis 1: Increase in the proportion of adult females

Assuming no intrinsic change in average cattle size through time, an increase in the proportion of the smaller adult females in the domestic cattle population might explain our observed data, and provide evidence for a change in herding strategies. For example, where milk production is the priority, a herder’s objective will be to ensure a large supply of lactating adult females. Hence, young males will often be culled once the milk yield is assured, which in unimproved African breeds ranges from 139–259 days [61], leading to a higher rate of female survival through adulthood. This sort of ‘post-lactation’ slaughter peak has been identified in the mortality profile of Neolithic European cattle (e.g. [62–63]). Stable nitrogen isotopic analyses of bone and dentine collagen in modern and ancient samples furthermore suggests that Neolithic cattle were being slaughtered at the end of the mothers lactation period, around the calves weaning age, to assist the milk let-down reflex [63–65]. Our sex ratio data does not support an increase in the survival rate of adult females, in fact showing an overall decrease in the proportion of adult females during the Late Neolithic. Analysis using the positively identified male and female osteometric measurements also contradicts this hypothesis by showing that both sexes underwent a size reduction, indicating a population-level phenotypic adaptation, rather than simply a shift in the sex ratio. Hence, the observed size diminution does not appear to be the result of an increase in the proportion of adult females in the population, although this does not negate dairying practices, nor does it refute a change in the rate of intensification of dairying over time.

#### Hypothesis 2: An increase in the proportion of reproductive sub-adults in the population resulted in the offspring achieving smaller adult size

The age and time of year at which animals give birth can have a significant impact on the size of their offspring. Some species, for example domestic cows and pigs, do not experience seasonal anoestrus and can therefore breed throughout the year, although their reproductive performance will ultimately be influenced by nutritional factors [66]. As a consequence, herders can more easily modulate the reproductive strategy of these animals in order to accommodate changes in the availability of forage or in response to other environmental effects.

Our analysis of the demographic structure of cattle herds reveals a significant increase in the proportion of sub-adults in the population during the Neolithic, synchronous with the decrease in adult size. Because the rate of body growth significantly slows at maturity (3–4 years in cattle), a strategy that maximises meat production will avoid retaining surplus stock beyond the sub-adult stage [67]. Consequently, there would be fewer reproductive adults and a greater proportion of reproductive sub-adults, resulting in potential lower birth weights due to the physiological and morphological constraints of giving birth before having reached adult body size [2–3].

Intensifying meat production is also suggested by an increase in the relative proportions of domestic pig over time [68], which is typically associated with an intensification of animal production [69]. Cattle, meanwhile, clearly play a central role in the Neolithic livestock economy of Central and northwest Europe [70], and are consistently well represented throughout the Neolithic and Early Bronze Age suggesting that any indication of intensification is likely to be observed in another aspect of their herding regime e.g. in body mass, milk production etc. We propose that the apparent increase in the proportion of sub-adults, and the decline in cattle body mass are indicative of an underlying change in the herding economy over time, which has a greater emphasis on meat productivity. Recent studies [71–72], have identified an increase in human population levels following the introduction of agriculture in the local Early Neolithic followed by a decline towards the end of the Middle Neolithic, and in some cases a secondary population increase during the later Neolithic or Early Bronze Age. This boom-bust pattern in regional population levels would have had major implications for the agro-pastoral systems of the time, leading to changes in the demands on animal productivity and input of labour, which may have unintentionally led to the size decrease observed here in Neolithic cattle.

Another factor, which we have not formally addressed here, is how these broad-scale changes in herding strategies relate to other forms of environmental modification, such as deteriorating pasture conditions. The practice of leaf foddering, as a means of providing dietary compensation has been well documented at Middle Neolithic sites in Switzerland and Denmark [73–74], the Paris Basin [75], and in southern France [76], and may be symptomatic of a change in the availability of nutrient-rich pasture. Similarly, evidence for slash-and-burn cultivation in the Late Neolithic in central Europe [77], would have allowed agriculture to expand into less suitable regions, increasing the availability of lower-quality feed from fallow grazing. As body mass is correlated with forage requirements due to calf weight being negatively affected by low nutrient intake in the gestating parent [78], one possible direction for further research would be to investigate links between changing cattle size, regional population pressures and deteriorating pasture conditions.

## Conclusion

Ultimately, the exact cause of the observed size decrease remains a puzzle, open to further investigation, requiring high-resolution archaeological and palaeoenvironmental data, such as detailed age-at-death profiles, and isotopic data to assess changes in birth seasonality (e.g. [79]). Nonetheless, our analysis provides compelling confirmation of a continental-wide post-domestication phenotypic adaptation, showing a size reduction of c.33% in Neolithic domestic cattle. Importantly, this trend is not observed in other domestic species, which may be due to the greater input of labour required in cattle, or a shift in their differential social status, i.e. from being a predominantly prestige resource during the Early Neolithic to a purely economic resource by the end of the Neolithic. Furthermore, we provide evidence of broad scale changes in the cattle herding strategies of Neolithic farmers, particularly an increase in the number of sub-adults in the death assemblage, which may be related to intensifying meat production. This occurs in parallel with an increase in the exploitation of other high meat yielding animals, such as the domestic pig, and could reflect a form of intensification driven by higher human populations levels.

## Supporting Information

S1 Table Relative frequencies of male, female and castrate bones per site phases identified using osteometric and morphological criteria. (CSV)

S2 Table Mean Log Size Index and Standard deviation per site phase for each species, including associated sample size, sitename, estimated mean date, period and cultural affiliation. (CSV)

## References

[1] Garel M, Cugnasse JM, Maillard D, Gaillard JM, Hewison MAJ, Dubray D. Selective harvesting and habitat loss produce long-term life history changes in a mouflon population. Ecol. Appl. 2007;17:1607–1618. 10.1890/06-0898.1 17913127

[2] Ozgul A, Tuljapurkar S, Benton TG, Pemberton JM, Clutton-Brock TH, Coulson T. The dynamics of phenotypic change and the shrinking sheep of St Kilda. Science 2009;325: 464–467. 10.1126/science.1173668 19574350 PMC5652310

[3] Ozgul A, Childs DZ, Oli MK, Armitage KB, Blumstein DT, Olson LE, et al Coupled dynamics of body mass and population growth in response to environmental change. Nature 2010;466: 482–487. 10.1038/nature09210 20651690 PMC5677226

[4] Gardner JL, Peters A, Kearney MR, Joseph L, Heinsohn R. Declining body size: a third universal response to warming? Trends Ecol. Evol. 2011;26, 6: 285–291. 10.1016/j.tree.2011.03.005 21470708

[5] Giroux MA, Tremblay JP, Anouk Simard M, Yoccoz NG, Côté SD. Forage-mediated density and climate effects on body mass in a temperate herbivore: a mechanistic approach. Ecology 2014; 95:1332–1340. 10.1890/13-0956.1 25000764

[6] Gingerich PD. Rates of evolution on the time scale of the evolutionary process. Genetica 2001;112–113: 127–144. 10.1007/978-94-010-0585-2_9 11838762

[7] Kinnison MT, Hendry NG. The pace of modern life II: from rates of contemporary microevolution to pattern and process. Genetica 2001;112–113, 145–164. 10.1007/978-94-010-0585-2_10 11838763

[8] Grant PR, Grant BR. Unpredictable evolution in a 30-year study of Darwin’s finches. Science 2002; 296: 707–711. 10.1126/science.1070315 11976447

[9] Estes S, Arnold SJ. Resolving the paradox of stasis: models with stabilizing selection explain evolutionary divergence on all timescales. Am. Nat. 2007;169: 227–244. 10.1086/510633 17211806

[10] Riesberg LH, Widmer A, Michele Arntz A, Burke JM. Directional selection is the primary cause of phenotypic diversification. PNAS 2002; 99,19: 12242–12245. 10.1073/pnas.192360899 12221290 PMC129429

[11] Boessneck J, Von Den Driesch A, Meyer-Lemppenau U & Wechsler Von Ohlen E. Die Tierknochenfunde aud dem Oppidum von Manching. Wiesbaden: Die Ausgrabungen in Manching 6; 1971.

[12] Bökönyi S. History of domestic animals in central and eastern Europe Budapest: Akadémiai Kiadó; 1974.

[13] Uerpmann HP. Metrical analysis of faunal remains from the Middle East In Zeder M, Meadow R, editors. Approaches to faunal analysis in the Middle East. Peabody Museum Bulletin 1 Cambridge: Harvard University Press; 1978 pp. 41–45

[14] Meadow RH. Osteological evidence for the process of animal domestication In: Clutton-Brock J. editor. The walking larder: Patterns of domestication, pastoralism, and predation. Unwin Hyman: London 1989 pp. 80–90.

[15] Zohary D, Tchernov E, Horwitz LK. The role of unconscious selection in the domestication of sheep and goats. J. Zool. 1998; 245: 129–135.

[16] Boessneck J, Von Den Driesch A. Die Tierknochenfunde aus der Neolithischen Siedlung auf dem Fikirtepe bei Madiky am Marmarameer. München: Institut für Palaeoanatomie, Domestikationsforschung und Geschichte der Tiermedizin der Universität München; 1978.

[17] Ijzereef GF. Bronze Age animal bones from Bovenkarspel. The excavation at Het Valkje. Nederlanse oudheden 10. Project Noord-Holland 1. Amersfoort: ROB; 1981.

[18] Méniel P. Contribution à l’histoire de l’élevage en Picardie du Néolithique à la fin de l’âge du Fer Amiens: Revue Archéologique de Picardie; 1984.

[19] Poplin F, Poulain T, Méniel P, Vigne JD, Geddes D, Helmer D. Les débuts de l’élevage en France In Demoule JP, Guilaine J, editors. Le néolithique de la France. Hommage à G. Bailloud Paris: Picard; 1986 p. 37–51.

[20] Vigne JD. Les Mammifères post-glaciaires de Corse, étude Archéozoologique (XXVIe supplement aé Gallia Préhistoire) Paris: Éditions du Centre National de la Recherche Scientifique; 1988.

[21] Tresset A. Early husbandry in Atlantic areas. Animal introductions, diffusion of techniques and native acculturation at the north-western margin of Europe. In Henderson J, editor. The Prehistory and early History of Atlantic Europe. British Archaeological Reports. International Series; 2000. pp. 17–32

[22] Lasota-Moskalewska A. Morphotic changes of domestic cattle skeleton from the Nelithic age to the beginning of the Iron Age. Wiadomści Archeologiczne 1980;45: 119–167.

[23] Schibler J, Schlumbaum A. Geschichte und wirtschaftliche Bedeutung des Hausrindes (Bos taurus L.) in der Schweiz von der Jungsteinzeit bis ins fruhe Mittrlalter. Schweiz Arch Tierh. 2007;149: 23–9. 10.1024/0036-7281.149.1.23 17243447

[24] Lyman RL, VanPool TL. Metric data in archaeology: a study of intra-analyst and inter-analyst variation. Am. Antiq. 2009;74: 485–504.

[25] Breslawski RP, Byers DA. Assessing measurement error in paleozoological osteometrics with bison remains. J. Arch. Sci. 2015;53: 235–242.

[26] Von den Driesch A. A guide to the measurement of animal bones from archaeological sites. Cambridge, Mass: Peabody Museum of Archaeology and Ethnology; 1976.

[27] Meadow RH. The use of size index scaling techniques for research on archaezoological collections from the Middle East In: Historia Animalium ex Ossibus. Festschrift für Angela von den Driesch zum 65. Geburtsta. Internationale Archäologie, Bd 8: Studia honoraria Rahden, Westfalia: Verlag Marie Leidorf; 1999 pp. 285–300

[28] Vigne JD, Helmer D, Peters J. New archaeozoological approaches to trace the first steps of animal domestication: general presentation, reflections and proposals In Vigne JD, Helmer D, Peters J, editors. First steps of animal domestication. New archaeozoological approaches Oxford: Oxbow books; 2005 pp.1–16.

[29] Evin A, Cucchi T, Cardini A, Vidarsdottir US, Larson G, Dobney K. The long and winding road: identifying pig domestication through molar size and shape. J. Archaeol. Sci. 2013;40: 735–743

[30] Simpson GG, Roe A, Lewontin RC. Quantitative Zoology, revised edition. New York: Harcourt, Brace and World; 1960.

[31] Manning K, Timpson A, Colledge S, Crema E, Edinborough K, Kerig T, et al The chronology of culture: a comparative assessment of European Neolithic dating approaches. Antiquity 2014; 88: 1065–1080.

[32] Purugganan MD, Fuller DQ. Archaeological data reveal slow rates of evolution during plant domestication. Evolution 2010; 65(1): 171–183. 10.1111/j.1558-5646.2010.01093.x 20666839

[33] Gautier M, Faraut T, Moazami-Goudarzi K, Navratil V. Foglio M, Grohs C, et al Genetic and haplotypic structure in 144 European and African cattle breeds. Genetics 2007; 177: 1059–1070. 17720924 10.1534/genetics.107.075804 PMC2034613

[34] Lari M, Rizzi E, Mona S, Corti G, Catalano G, Chen K, et al The complete mitochondrial genome of an 11,450 year old aurochsen (Bos primigenius) from Central Italy. Evol. Biol. 2011;11: 32. 10.1186/1471-2148-11-32 PMC3039592 21281509

[35] Kaeuffer R, Coltman DW, Chapuis JL, Pontier D, Réale D. Unexpected heterozygosity in an island mouflon population founded by a single pair of individuals. Proc. B. Soc. B 2008; 274, 1609: 527–533. 10.1098/rspb.2006.3743 PMC1766376 17476773

[36] Luikart G, Gielly L, Excoffier L, Vigne JD, Bouvet J, Taberlet P. Multiple maternal origins and weak phylogeographic structure in domestic goats. PNAS 2001; 98(10): 5927–5932 11344314 10.1073/pnas.091591198 PMC33315

[37] Groenen MAM, Archibald AL, Uenishi H, Tuggle CK, Takeuchi Y, Rothschild MF, et al Analyses of pig genomes provide insight into porcine demography and evolution. Nature 2012; 491: 393–398. 10.1038/nature11622 23151582 PMC3566564

[38] Sacks BN, Brown SK, Stephens D, Pederson NC, Wu JT, Berry O. Y Chromosome analysis of dingoes and Southeast Asian village dogs suggests a Neolithic continental expansion from Southeast Asia followed by multiple Austronesian dispersals. Mol. Biol. Evol. 2013; 30(5): 1103–1118. 10.1093/molbev/mst027 23408799

[39] Haldane JBS. The rate of mutation of human genes. Hereditas 1949; 35(1): 267–273.

[40] Hendry AP, Nosil P, Riesberg LH. The speed of ecological speciation. Funct. Ecol. 2007; 21(3): 455–464. 10.1111/j.1365-2435.2007.01240.x 19096732 PMC2605086

[41] Rowley-Conwy P, Gourichon L, Helmer D, Vigne JD. Early domestic animals in Italy, Istria, the Tyrrhenian Islands and southern France In Colledge S, Conolly J, Dobney K, Manning K, Shennan S, editors. The Origins and Spread of Domestic Animals in Southwest Asia and Europe. Walnut Creek: Leftcoast Press 2013 pp. 161–194.

[42] Trut L, Osinka I, Kharlamova A. Animal evolution during domestication: the domesticated fox as a model. Bioessays 2009; 31(3): 349–360. 10.1002/bies.200800070 19260016 PMC2763232

[43] Helmer D, Gourichon L, Monchot H, Peters J, Saña-Segui M. Identifying early domestic cattle from prepottery Neolithic sites on the middle Euphrates using sexual dimorphism In: Vigne JD, Peters J, Helmer D, editors. The First Steps of Animal Domestication. Oxford: Oxbow Books; 2005 pp 86–95.

[44] Bökönyi S. Archeological problems and methods of recognizing animal domestication In: Ucko PJ & Dimbleby GW. The domestication and exploitation of plants and animals. London: Duckworth 1969 pp. 219–229.

[45] Price EO. Behavioral aspects of animal domestication. Q. Rev. Biol. 1984;59: 1–32.

[46] Peters J, Helmer D, von den Driesch A, Saña Segui M. Early Animal Husbandry in the Northern Levant. Paléorient 1999; 25, 2: 27–48.

[47] Colledge S, Conolly J, Dobney K, Manning K, Shennan S, editors. The Origins and Spread of Domestic Animals in Southwest Asia and Europe. Walnut Creek: Leftcoast Press; 2013.

[48] Meadow RH. Animal domestication in the Middle East: A view from the eastern margin In Clutton-Brock J, Grigson C, editors. Animals and archaeology, volume 3: Early herders and their flocks. BAR International Series Oxford: Archaeopress; 1984 pp. 309–337.

[49] Hongo H, Meadow R. Faunal remains from Prepottery Neolithic levels at Çayönü, southeastern Turkey: a preliminary report focusing on pigs (Sus sp.) In Marshkour M, Choyke AM, Buitenhuis H, Poplin F, editors. Archaeozoology of Southwest Asia IV. Groningen: ARC-Publications; 2000 pp. 121–140.

[50] Albarella U, Dobney K, Rowley-Conwy P. The domestication of the pig (Sus scrofa): new challenges—and approaches In Zeder M, Bradley D, Emshwiller E, Smith B, editors. Documenting Domestication: new genetic and archaeological paradigms. Berkeley: University of California Press; 2006 pp.209–27.

[51] Bollongino R, Burger J, Powell A, Mashkour M, Vigne J-D, Thomas MG. Modern Taurine Cattle Descended from Small Number of Near-Eastern Founders. Mol. Biol. Evol. Letter 2012: 29(9):2101–2104. 10.1093/molbev/mss 22422765

[52] Edwards CJ, Bollongino R, Scheu A, Chamberlain A, Tresset A, Vigne JD, et al Mitochondrial DNA analysis shows a Near Eastern Neolithic origin for domestic cattle and no indication of domestication of European aurochs. Proc. R. Soc. B, 2007;274: 1616 1377–1385. 10.1098/rspb.2007.0020 PMC2176208 17412685

[53] Bollongino R, Elsner J, Vigne JD, Burger J. Y-SNPs do not indicate hybridization between European aurochs and domestic cattle. PlosOne 2008;3(10) e3418 10.1371/journal.pone.0003418 PMC2561061 18852900

[54] Bergmann C. Über die Verhältnisse der Wärmeökonomie der Thiere zu ihrer Grösse. Göttinger Studien 1847; 3(1): 595–708.

[55] Audoin-Rouzeau F. La taille des animaux d’élevage é l’époque romaine et leur exportation. In Chevallier R, editor. Homme et animal dans l’antiquité romaine, Actes du colloque de nantes 1991. Tours: Centre de Recherche. 1995. pp. 79–100.

[56] Schlumbaum A, Stopp B, Breuer G, Rehazek A, Blatter R, Turgay M, et al Combining archaeozoology and molecular genetics: the reason behind the changes in cattle size between 150BC and 700AD in Northern Switzerland. Antiquity 2003;77, 298: Project Gallery.

[57] Albarella U, Johnstone C, Vickers K. 2008. The development of animal husbandry from the late Iron Age to the end of the Roman period: a case study from south-east Britain. J. Archaeol. Sci. 2008; 20: 1–21. 10.1016/j.jas.2007.11.016

[58] MacKinnon M. Cattle “breed” variation and improvement in Roman Italy: connecting the zooarchaeological and ancient textual evidence. World Archaeol. 2010; 42(1): 55–73. 10.1080/00438240903429730

[59] Colominas L, Schlumbaum A, Saña M. The impact of the Roman Empire on animal husbandry practices: study of the changes in cattle morphology in the north-east of the Iberian Peninsula through osteometric and ancient DNA analyses. Archaeol. Anthropol. Sci. 2014; 6: 1–16. 10.1007/s12520-013-0116-9

[60] Luff RM. Animal bones from excavations in Colchester, 1971–85. Colch. Archaeol. Rep. 12. Colchester: Colchester Archaeological Trust Ltd; 1993.

[61] Dahl G, Hjort A. Having herds: Pastoral herd growth and household economy Stockholm Studies in Social Anthropology 2 Department of Social Anthropology, University of Stockholm, Stockholm, Sweden; 1976.

[62] Tresset A. Le rôle des relations homme-animal dans l’ évolution économique et culturelle des sociétés des Ve-IVe millénaires en Bassin parisien: Approche ethno-zootechnique fondée sur les ossements animaus. PhD thesis, Université Paris-I Panthéon-Sorbonne. 1996.

[63] Gillis R, Bréhard S, Bălăşescu A, Ughetto-Monfrin J, Popovici J, Vigne JD, et al Sophisticated cattle dairy husbandry at Borduşani-Popină (Romania, fifth millennium BC): the evidence from complementary analysis of mortality profiles and stable isotopes. World Archaeol. 2014; 45:3: 447–472. 10.1080/00438243.2013.820652

[64] Balasse M. Keeping the young alive to stimulate milk production? Differences between cattle and small stock. Anthropozoologica 2003; 37: 3–37.

[65] Balasse M, Tresset A. Early weaning of Neolithic domestic cattle (Bercy, France) revealed by intra-tooth variation in Nitrogen isotope ratios. J. Arch. Sci. 2002; 29: 853–9.

[66] Reinhardt C, Reinhardt A, Reinhardt V. Social behavior and reproductive performance in semi-wild Scottish Highland cattle. Appl. Anim. Behav. Sci. 1986; 15: 125–136. 10.1016/0168-1591(86)90058-4

[67] Payne S. Kill-off patterns in sheep and goats: The mandibles from Aşvan Kale. Anatol. Stud. 1973; 23: 281–303. DOI: 10.2307/3642547

[68] Schibler J. 2013. Zooarchaeological data from Late Mesolithic and Neolithic sites in Switzerland In Colledge S, Conolly J, Dobney K, Manning K, Shennan S, editors. The Origins and Spread of Domestic Animals in Southwest Asia and Europe. Walnut Creek: Leftcoast Press 2013 pp. 253–269.

[69] Fraser DG. 2005 *Animal welfare and the intensification of animal production*: *an alternative interpretation* (Vol. 2). Food & Agriculture Organisation

[70] Manning K, Downey SS, Colledge S, Conolly J, Stopp B, Dobney K, et al 2013 The origins and spread of stock-keeping: the role of cultural and environmental influences on early Neolithic animal exploitation in Europe. Antiquity 87 (338): 1046–1059. 10.1017/S0003598X00049851

[71] Shennan S, Downey SS, Timpson A, Edinborough K, Colledge S, Kerig T, et al Regional population collapse followed initial agriculture booms in mid-Holocene Europe. Nat. Commun. 2013; 4: 2486 10.1038/ncomms3486 24084891 PMC3806351

[72] Timpson A, Colledge S, Crema E, Edinborough K, Kerig T, Manning K, et al Reconstructing regional demographies of the European Neolithic using ‘dates as data’: a new case-study using an improved method. J. Arch. Sci. 2014; 52: 549–557. 10.1016/j.jas.2014.08.011

[73] Rasmussen P. Leaf foddering in the earliest Neolithic agriculture. Evidence from Switzerland and Denmark. Acta Archaeol. 1989; 60: 71–85.

[74] Akeret Ö, Rentzel P. Micromorphology and plant macrofossil analysis of cattle dung from the Neolithic lake shore settlement of Arbon Bleiche 3. Geoarchaeology 2001; 16(6): 687–700. 10.1002/gea.1016

[75] Balasse M, Boury L, Ughetto-Monfrin J, Tresset A. Stable isotope insights (*δ* ^18^ O, *δ* ^13^C) into cattle and sheep husbandry at Bercy (Paris, France, 4^th^ millennium BC): birth, seasonality and winter leaf foddering. Environ. Archaeol. 2012; 17(1): 29–44. 10.1179/1461410312Z.0000000003

[76] Delhon C, Martin L, Argant J, Thiébault S. Shepherds and plants in the Alps: multi-proxy archaeobotanical analysis of Neolithic dung from La Grande Rivoire’ (Isère, France). J. Arch. Sci. 2008; 35: 89–97.

[77] Rösch M. Land use and food production in Central Europe from the Neolithic to the Medieval period: Change of landscape, soils and agricultural systems according to archaeobotanical data In Kerig T, Zimmerman A, editors. Economic Archaeology: From structure to performance in European Archaeology. Bonn: Habelt; 2013 pp. 109–128.

[78] Martin JL, Vonnahme KA, Adams DC, Lardy GP, Funston RN. Effects of dam nutrition on growth and reproductive performance of heifer calves. J. Anim. Sci. 2007; 85: 841–847. 10.2527/jas.2006-337 17085735

[79] Balasse M, Tresset A. Environmental constraints on the reproductive activity of domestic sheep and cattle: what latitude for the herder? Anthropozoologica 2007; 42(2): 71–88.

